# Targeting TNF and TNF Receptor Pathway in HIV-1 Infection: from Immune Activation to Viral Reservoirs

**DOI:** 10.3390/v9040064

**Published:** 2017-03-30

**Authors:** Sébastien Pasquereau, Amit Kumar, Georges Herbein

**Affiliations:** Department of Virology, University of Franche-Comte, University of Bourgogne-Franche-Comté (UBFC), CHRU Besançon, UPRES EA4266 Pathogens & Inflammation/EPILAB, SFR FED 4234, F-25030 Besançon, France; sebastien.pasquereau@univ-fcomte.fr (S.P.); amit.aiims2005@gmail.com (A.K.)

**Keywords:** TNF, TNF receptor, HIV-1, reservoirs, immune activation, anti-TNF therapy

## Abstract

Several cellular functions such as apoptosis, cellular proliferation, inflammation, and immune regulation involve the tumor necrosis factor-α (TNF)/TNF receptor (TNFR) pathway. Human immunodeficiency virus 1 (HIV-1) interacts with the TNF/TNFR pathway. The activation of the TNF/TNFR pathway impacts HIV-1 replication, and the TNF/TNFR pathway is the target of HIV-1 proteins. A hallmark of HIV-1 infection is immune activation and inflammation with increased levels of TNF in the plasma and the tissues. Therefore, the control of the TNF/TNFR pathway by new therapeutic approaches could participate in the control of immune activation and impact both viral replication and viral persistence. In this review, we will describe the intricate interplay between HIV-1 proteins and TNF/TNFR signaling and how TNF/TNFR activation modulates HIV-1 replication and discuss new therapeutic approaches, especially anti-TNF therapy, that could control this pathway and ultimately favor the clearance of infected cells to cure HIV-infected patients.

## 1. Introduction

Tumor necrosis factor-α (TNF) was first described as a glycoprotein induced in response to endotoxin (the old name of which was cachectin) and has the capacity to kill tumor cells [1]. Then, TNF was shown to be a T helper type 1 (Th1) cytokine produced by several cell types, including monocytes/macrophages and T-cells, and to be involved in proinflammatory responses like interleukin (IL)-1β and IL-6 [2]. TNF exerts its effects by binding as a trimer to two cell membrane TNF receptors (TNFRs), TNFR1 (55-kDa) and TNFR2 (75 kDa) [2]. In concert with chemokines, TNF and TNFRs are involved in the regulation of inflammatory processes like arthritis, of infectious diseases including HIV infection, and of malignancies, since chronic inflammation has been recognized to play a major role in carcinogenesis [3,4,5].TNF was first recognized at the protein level, followed by identification of its cDNA sequence [4,6,7]. TNFRs are composed of an extracellular domain common to all TNFR superfamily members, a typical cysteine rich motif, and intracellular domains with distinct motifs, allowing the accomplishment of various intracellular functions [8,9]. Death receptors are members of the TNFR superfamily characterized by a cytoplasmic region, the death domain, that enables the receptors to trigger pro-death signals within the cell [4]. TNFR1 possesses a death domain, and TNFR2 lacks one. This allows TNF to deliver distinct intracellular signals after binding to its receptors. We will discuss in this review how the binding of TNF to its receptors modulates the cellular activation, the interaction between human immunodeficiency virus 1 (HIV-1) proteins, and TNF/TNFR signaling, and the role of TNF/TNFRs in the modulation of the viral life cycle. In addition, we will address the impact of the TNF/TNFR pathway on the immune activation and the formation of viral reservoirs during the course of HIV-1 infection. The opportunity to modulate the TNF effect and the TNFR pathway by anti-TNF therapy and/or to stimulate viral reactivation by TNF or TNF inducers to purge the HIV-1 reservoir will be discussed.

## 2. TNF/TNFR Signaling

**Figure 1:**
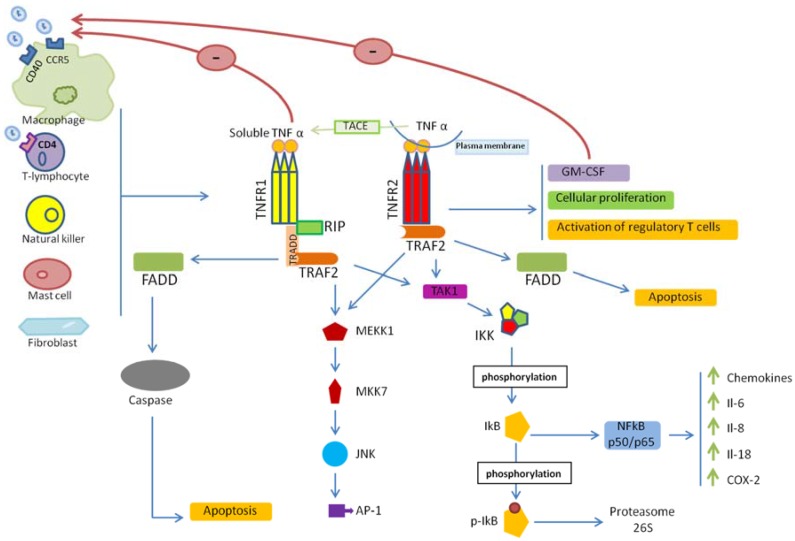
Tumor necrosis factor-α (TNF)/TNF receptor (TNFR)-mediated cell signaling. The binding of TNF to TNFRs leads to the recruitment of adaptor proteins—TNFR-associated death domain (TRADD), Fas-Associated death domain (FADD), TNF receptor associated factor (TRAF), and receptor interacting protein (RIP)—that activates several signaling cascades leading to the activation of transcription factors (NF-κB, AP-1 among others) and/or caspase cascades. COX-2: cyclooxygenase-2; FADD: Fas-associated death domain; GM-CSF: Granulocyte-macrophage colony-stimulating factor; IκB: NF-κB inhibitor; IKK: IκB kinase; Il: Interleukin; NFκB: Nuclear factor-κB; TACE: TNF-α converting enzyme; TRAF2: TNFR-associated factor 2; TRADD: TNFR-associated death domain.

Many cell types, especially immune cells, produce TNF in response to pathological conditions such as inflammation and infection, including: activated macrophages and T lymphocytes, natural killer (NK) cells, mast cells, and fibroblasts [4,8]. A membrane protein belonging to the ADAM (a disintegrin and metalloproteinase) family, the TNF-α converting enzyme (TACE) cleaves various membrane proteins including the TNF precursor, a 25 kDa plasma bound protein, into a 17 kDa soluble TNF form [10]. In their trimeric configuration, both membrane-bound and soluble TNF forms are functional when binding to their receptors. Although both TNF forms can bind to each of the two receptors, they display a dichotomic effect. The plasma membrane associated with 25 kDa TNF binds preferentially to the TNFR2, whereas the soluble 17 kDa TNF interacts with TNFR1 with high specificity [11,12]. Several intracellular pathways are triggered by TNF binding to its receptors, including the modulation of the apoptotic pathway, NF-κB stimulation, and the activation of p38 mitogen-activated protein kinases (MAPK), extracellular signal-regulated kinase (ERK), and c-Jun N-terminal kinase (JNK) [4,8] (Figure 1).

A 34 kDa adapter protein called TNFR-associated death domain (TRADD) interacts with the cytoplasmic death domain of TNFR1 through its own death domain in response to the binding of TNF to TNFR1 [13] (Figure 1). Further, a Fas-Associated death domain protein (FADD) binds to TRADD and activates apoptosis via the caspase cascade [14] (Figure 1). On the other hand, the sequential recruitment of receptor interacting protein (RIP), transforming growth factor (TGF)-β-activated kinase 1 (TAK1), and IκB kinase (IKK) by the TNF receptor associated factor 2 (TRAF2) forms a complex that interacts with TRADD [8,15,16]. The phosphorylation of IκB by IKK and its subsequent degradation in the proteasome leads to NF-κB translocation into the nucleus, where it binds to the promoters of genes involved in inflammation, such as TNF, IL-6, IL-8, IL-18, chemokines, and cyclooxygenase-2 (COX-2) genes [4,8,15,17] (Figure 1). In addition to stress and growth factors, cellular proliferation can be triggered by TNF binding to TNFR1, followed by activation of the following axis; TRADD, TRAF2-RIP, mitogen-activated protein kinase kinase kinase 1(MEKK1), mitogen-activated protein kinase kinase 7 (MKK7), and JNK, ultimately leading to the activation of the AP-1 transcription factor [4,8,18] (Figure 1). Although TNFR2 lacks a death domain, it interacts with TRAF2 and can activate the transcription factors NF-κB and AP-1 (Figure 1). Cell proliferation and survival, activation of regulatory T-cells, and the induction of granulocyte-macrophage colony-stimulating factor (GM-CSF) secretion can be elicited through TNF/TNFR2 signaling [19,20,21].

## 3. TNF/TNFRs and HIV-1 Entry

The role of TNF in HIV-1 entry has been extensively described, especially in primary human macrophages (Figure 1) [22]. In vitro, the primary macrophages infected by HIV-1 or treated with HIV-1 envelope protein gp120 release TNF in culture supernatants [23]. A delayed detection of HIV DNA long terminal repeat (LTR) up to 72 h has been observed in macrophages pre-treated by TNF parallel to the downregulation of the CD4 receptor [22,23,24,25,26]. Therefore, one explanation for the inhibition of HIV entry into permissive cells by TNF might be the down regulation of the expression of the HIV CD4 receptor and the C–C chemokine receptor type 5 (CCR5) coreceptor on the cell surface [24]. In fact, CCR5 downregulation on the cell surface results from the secretion of GM-CSF following the stimulation of macrophages with TNF, which will ultimately result in the inhibition of the entry of CCR5-dependent HIV-1 strains [25]. In addition, the binding of TNF to TNFR2 activates NF-κB and favors enhanced secretion of HIV suppressive factors, such as macrophage inflammatory protein-1α (MIP-1α), MIP-1β, and regulated on activation, normal T-cell expressed and secreted (RANTES). These factors also downregulate CCR5 on the cell surface [27,28,29,30,31], indicating that the TNF inhibition of HIV entry is mediated through TNFR2 but not TNFR1. However, the inhibition of viral entry is not related to the CD4 downregulation in macrophages. Both surface and total CD4 are downregulated in macrophages at the level of transcription after TNF pretreatment for one to five days [23,24]. However, after TNF pretreatment for 2 h, a 75% decrease in virus entry is observed, although the levels of surface CD4 expression are not yet modified. In addition, the surface CD4 downregulation in TNF-treated macrophages is mediated through TNFR1 but not through TNFR2 [22,23,24,26]. Therefore, the inhibition of HIV-1 entry into macrophages by TNF is mediated through the TNF-R2 but not through TNF-R1 and is independent of the modulation of surface CD4 expression. Altogether, the downregulation of the CCR5 co-receptor is the main mechanism that inhibits HIV entry into macrophages treated with TNF and is not related to the downregulation of the CD4 receptor. However, we cannot exclude that the inhibition of the HIV-1 entry into macrophages could also result from post-binding steps, which have still to be identified.

## 4. TNF/TNFR Signaling in HIV-1 Infection

**Figure 2:**
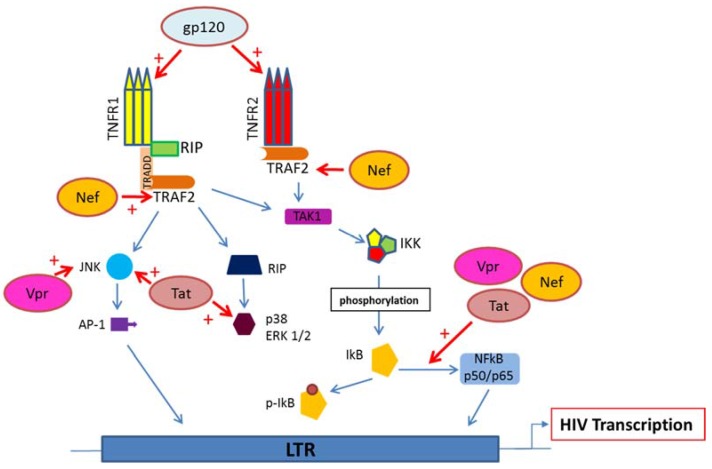
The TNF/TNFR pathway is targeted by human immunodeficiency virus 1 (HIV-1) proteins. HIV-1 proteins viral protein R (Vpr), trans-activator of transcription (Tat), negative regulatory factor (Nef), and envelope glycoprotein gp120 interfere with the TNF/TNFR pathway to enhance HIV transcription in infected cells.

Several intracellular signaling pathways, including NF-κB, JNK, and p38 MAP kinase pathways, are triggered by TNF (Figure 1) [32]. Diverse cellular functions are regulated by the members of the p38 family, consisting of p38α, p38β, p38γ, and p38δ, through the phosphorylation of transcription factors such as activator protein 1 (AP-1), activating transcription factor 2 (ATF-2), and cAMP response element binding protein(CREB) [33]. The first isoform discovered for its role in endotoxin-induced inflammatory response and osmotic shock is p38α [33,34]. Independent or synergistic activation of NF-κB and p38 by TNF can be observed [35,36]. Specific signaling pathways leading to the activation of either pro-survival mediators or effectors of cell death can be observed in response to TNF stimulation. By binding to TNFR, TNF activates NF-κB, which promotes cell survival, JNKs, and caspases, which favor cell death. Therefore, an exquisite crosstalk between the two pathways will decide the cellular fate [37]. In addition, in HIV-1 infected cells, TNF stimulation will favor the binding of p65/p50 NF-κB and AP-1 complexes to their respective transcription factor binding sites present in the HIV-1 long terminal repeat (LTR) promoter. Thus, TNF-mediated translocation of NF-κB to the nucleus, followed by LTR stimulation, fuels HIV-1 production in chronically infected T-cell lines and promonocytic cell lines, such as the ACH2 and U1 cell lines, respectively [38,39,40,41,42]. Although TNF could be a major driver of HIV-1 transcription early in the disease, later on, during the progression of the disease, a T helper Th1/Th2 shift has been described [43]. Since Th2 cytokines are mostly immunosuppressive with decreased levels of TNF production at advanced stages of the disease, HIV-1 replication could be sustained by the production of some HIV proteins that mimic TNF activity. We and others reported previously that several HIV-1 proteins, especially viral protein R (Vpr), trans-activator of transcription (Tat), negative regulatory factor (Nef), and envelope glycoprotein gp120, activate signaling pathways that are also triggered by TNF, especially in macrophages (Reviewed in [44]) (Figure 2).

## 5. HIV Proteins Interaction with TNFR and Downstream Signaling Pathways

Vpr encodes a 96-amino acid, 14 kDa protein, which is conserved among HIV-1, HIV-2, and simian immunodeficiency virus. Multiple cellular functions are targeted by Vpr, including the induction of cell cycle arrest in the G2 phase, transactivation of the LTR promoter, nuclear import of preintegration complexes, and modulation of apoptosis. Both pro- and anti-apoptotic roles of Vpr have been described. Vpr triggers apoptosis in infected T-cells through activation of caspases 3/7, 8, and 9 [45,46] and by blocking NF-κB activation in myeloid cells [47]. In contrast, Vpr expression in the Jurkat cell line has been reported to enhance resistance to TNF-induced apoptosis [48]. In addition to these functions, Vpr triggers the mitochondrial dysfunction [49,50]. Vpr is critical for HIV replication in non-dividing cells, e.g., macrophages [44], but is not required for replication in T-cells. Like TNF, recombinant Vpr activates NF-κB, AP-1, and JNK in primary macrophages and monocytoid U937 cells, resulting in the stimulation of HIV-1 LTR and the subsequent enhancement of viral replication [44,51]. Vpr-dependent activation of NF-κB has also been reported in primary T-cells [52]. In addition, HIV production is increased by Vpr through toll-like receptor 4 (TLR4) and IL-6 secretion [50]. Recently, Vpr has been reported to enhance TNF production by HIV-1-infected T-cells, implicating the cellular proteins TAK1 and damage-specific DNA binding protein 1 (DDB1) in this phenomenon [53]. TNF produced by infected cells activates NF-κB in bystander cells and favors viral reactivation from the HIV-1 reservoir [53]. Also, enhanced stimulation of HIV-1 LTR results from a synergistic effect of Vpr and Tat [54]. A few reports indicate that in some cases Vpr can inhibit NF-κB activation. For example, the inhibition of NF-κB mediated gene expression results from a synergistic action of Vpr and glucocorticoid receptors via a pathway involving the suppressor of poly (ADP-ribose) polymerase (PARP)-1 nuclear trafficking in response to TNF [47,55]. Thus, the respective roles of Vpr and TNF on cellular functions and HIV-1 replication are complex and depend on the cell type involved.

A close interplay between TNF and HIV-1 Nef is present throughout the progression of the disease [56]. The multifunctional 27kDa myristoylated Nef protein triggers NF-κB activation in macrophages and monocytoid U937 cells, favoring the HIV LTR activation in vitro [57,58], and the transcription of several genes coding for proinflammatory cytokines and chemokines such as TNF, IL-6, MIP-1α, and MIP-1β [59,60,61]. The phosphorylation of several key-signaling molecules, including α/β subunits of IκB kinase, ERK1/2, JNK, and p38, is triggered in macrophages treated with recombinant Nef in vitro [60]. Moreover, Nef and TNF synergistically stimulate the HIV-1 replication in monocytic cells and primary macrophages [62], and Nef directly interacts with several TRAF proteins (TRAF2, TRAF5, TRAF6) to enhance the HIV-1 replication in macrophages [63]. In addition to a plasma membrane(PM)-associated TNF shedding, the secretion of vesicular TNF endosomes has been recently described as an alternative TNF secretion mechanism, where Nef-mediated routing of the TNF alpha-converting enzyme (TACE) ADAM17 into Rab4+ early endosomes and the Rab27+ secretory pathway leads to intracellular proTNF cleavage and secretion of vesicular TNF endosomes [64]. For the vesicular TNF endosomal trafficking of disintegrin and metalloproteinase domain-containing protein 17 (ADAM17), neurogenic locus notch homolog protein 1 (Notch1) is required [64]. In fact, in exosomes released from cells infected with viruses other than HIV, the presence of viral components has been reported including, among others, components of Epstein-Barr virus, cytomegalovirus, herpes simplex virus, and hepatitis C virus [65]. Although the role of vesicular TNF has been less studied so far than its soluble TNF counterpart, T-cell activation, viral replication, and viral reactivation from HIV reservoir are enhanced by exosomes of HIV-1-infected cells through a Nef- and TNF-dependent mechanism [66,67]. In addition, exosomes from HIV-1-infected cells containing trans-activation response (TAR) RNA stimulate the production of TNF in primary macrophages [68]. Finally, exposure to Nef-containing exosomes released from transfected T-cell lines increases CD4+ T-cell apoptosis [57].

The HIV-1 Tat protein is critical for the elongation process during viral transcription, but also activates NF-κB, JNK, p38, and ERK1/2 pathways and thereby could mimic the effect of TNF on cellular gene expression [44,69] (Figure 2). Both MyD88 and TIR-domain-containing adapter-inducing interferon-β (TRIF) axes recruit the TLR4 pathway, following stimulation by Tat, and lead to the activation of protein kinase C beta2 isoform, MAP kinase, and NF-κB, thus resulting in enhanced TNF production in monocytes [70].

The production of TNF, but also of IL-6, IL-10, and interferon γ, has been reported after the interaction of gp120 with CD4 (Reviewed in [71]). The production of TNF is triggered by gp120 in peripheral blood mononuclear cells isolated from HIV-1 infected patients [72,73,74], in macrophages treated with gp120 in vitro [75], and in animal models [76]. The PI3K and MAPK pathways are involved in the increase of TNF production, which is triggered by HIV gp120 [75,77]. In HIV-infected patients, a positive correlation between the levels of gp120 and the expression profiles of proinflammatory cytokines, including TNF, has been reported [78].

The interaction between macrophage-membrane bound TNF and TNFR2 present on CD8+ T- cells triggers CD8+ T-cell apoptosis [79]. Chronic inflammation in HIV-1 infection is characterized by the detection of soluble markers of immune activation, including, among others, neopterin, β2 microglobulin, soluble CD30, TNF, and the soluble TNFR2 (sTNFR2) [80]. In symptomatic HIV-infected patients, increased TNF levels are detected in the serum, and elevated levels of sTNFR2 are predictive of disease progression [81].

## 6. Anti-TNF Medications

In several chronic inflammatory diseases, such as rheumatoid arthritis, ankylosing spondylitis, juvenile arthritis, psoriatic arthritis, Crohn’s disease, and ulcerative colitis, the blockade of TNF signaling is commonly used. Thus, medications such as antibodies or receptors, which target the TNF/TNFR pathway for treating various inflammatory diseases, are now in preclinical or clinical phases of development [82,83]. In 1998, the human/murine chimeric antibody, infliximab, was approved. It binds with high affinity to both the soluble and the membrane-bound TNF. It was then followed by etanercept, a TNFR2-fragment cristallizable region (Fc) fusion protein, that acts like a decoy to block TNF [84]. In 2002 and 2009, the fully human antibodies adalimumab and golimumab, which bind to TNF, were approved. Moreover, certolizumab pegol was approved in 2008. This anti-TNF monoclonal antibody contains a PEGylated Fab fragment, which targets TNF, with the advantage of being administrated to pregnant women. Thus, pregnant women with autoimmune diseases and in need of anti-TNF therapy can be treated with certolizumab pegol, since polyethylene glycol does not cross the placenta [85]. Additionally, the cells involved in inflammation could be lysed by adalimumab and infliximab, since both medications trigger apoptosis and cell cycle arrest in transmembrane TNF-expressing Jurkat T-cells. This could result in immune suppression, thus improving the course of inflammatory diseases, such as Crohn’s disease, but with a risk of reactivation of latent tuberculosis in infected patients [82]. This negative effect can be prevented by appropriate screening, and HIV-infected patients should be adequately vaccinated when possible and closely monitored for early signs of infection. When serious infections occur, withdrawal of anti-TNF therapy may be necessary until the infection has been identified and properly treated.

## 7. Anti-TNF Therapy and Control of Immune Activation in HIV-1 Infection

**Figure 3:**
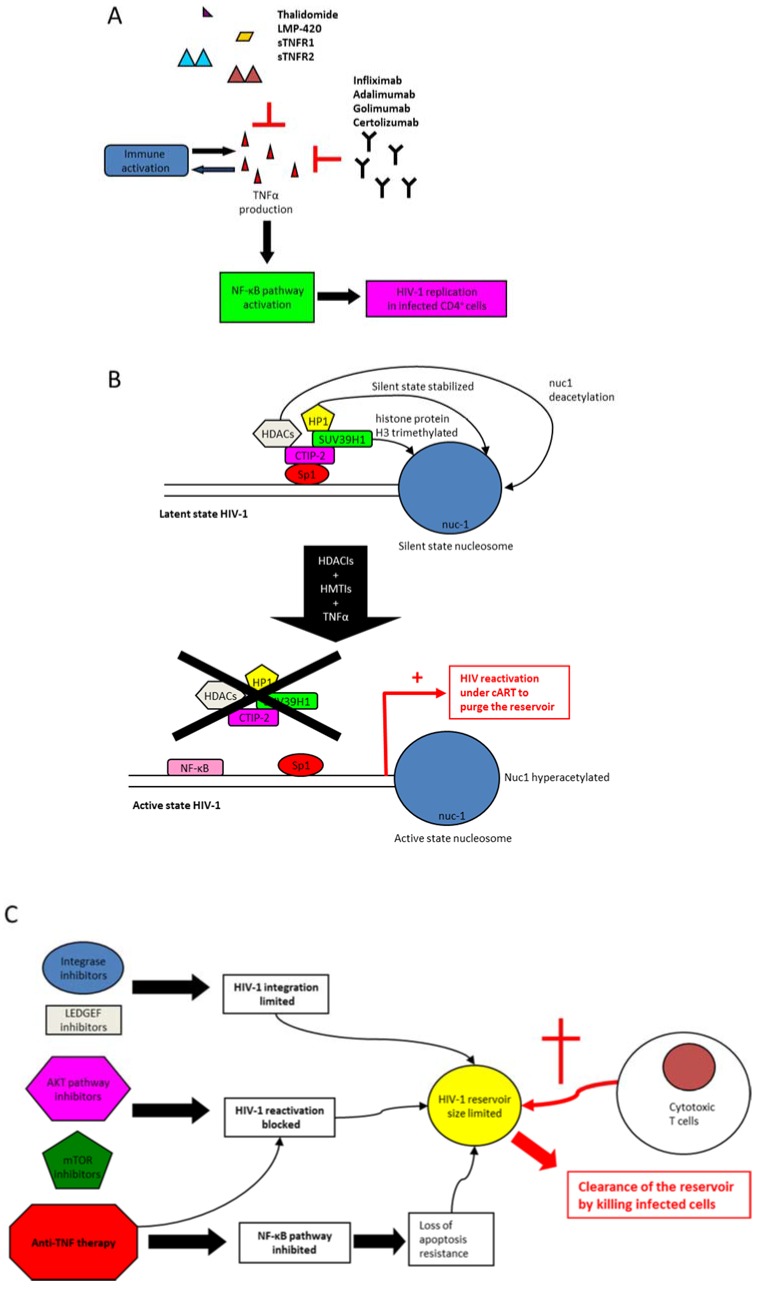
Potential TNF-based therapies to control HIV-1 infection. (**A**) Anti-TNF therapy against immune activation. Anti-TNF therapy limits immune activation triggered by HIV-1 infection. (**B**) TNF stimulation to enhance viral reactivation: the ‘shock and kill’ strategy. During HIV-1 latency, nuc-1 nucleosome is hypoacetylated. Coup-TFI interacting protein 2 (CTIP-2) interacts with Sp1, switching nuc-1 from transcriptionally active to a repressive state. Furthermore, CTIP-2 recruits histone deacetylase inhibitors (HDACs) that deacetylate the nuc-1 nucleosome. Suppressor of variegation 3-9 homolog 1 (SUV39H1) adds a trimethylation mark onto the histone protein H3. Furthermore, the heterochromatin protein 1 (HP1) protein stabilizes the nuc-1 in a transcriptionally silent state. The combination of TNF with HDAC inhibitors (HDACIs) or histone methyltransferase (HMT) inhibitors (HMTIs) can disrupt the HIV-1 latency. TNF and HDACIs can trigger the activation of transcriptional activators such as NF-κB (p50/p65 heterodimer). HDACIs prevent the formation of heterochromatin, resulting in nuc-1 hyperacetylation and remodeling, thereby alleviating the HIV transcriptional block. The use of TNF, HDACIs, and HMTIs enhances HIV-1 LTR transcription and could participate in the purge of the HIV-1 reservoir under combination antiretroviral therapy (cART). (**C**) Anti-TNF therapy to limit the size of the HIV-1 reservoir; the ‘block and lock’ strategy. Integrase inhibitors, lens epithelium-derived growth factor (LEDGF) inhibitors, mammalian target of rapamycin (mTOR) inhibitors and protein kinase B (AKT) inhibitors limit both HIV-1 integration and reactivation, thereby blocking and locking a limited amount of provirus, which then could be cleared by anti-HIV cytotoxic T-cells (CTLs). nuc-1: single nucleosome.

At all stages of HIV-1 infection, increased amounts of TNF can be detected either in the plasma or in the tissues [38,86,87]. Even in combination antiretroviral therapy (cART) treated patients with undetectable viremia, immune activation is still present along with increased levels of plasma TNF [88,89,90,91]. In HIV-infected patients, the increased viral replication and depletion of CD4+ T-cells could result from enhanced TNF expression [92,93]. In addition, TNF impairs CD4+ T-cell-mediated immunological control in chronic viral infection [94]. Therefore, in addition to the inhibition of viral replication by cART, new therapeutic approaches, which control TNF production, could be helpful to curtail immune activation in HIV-infected patients. Thus, increased levels of TNF in HIV infection could be counteracted by anti-TNF therapy (Figure 3A).

Moreover, the release of soluble TNF receptors (sTNFR1 and sTNFR2), following the membrane-bound TNFR shedding by proteolytic cleavage, can compete with the membrane-bound TNFRs for binding to TNF, thus inhibiting its activity. In addition, the cells could be transiently desensitized to TNF action, due to the decrease in the number of membrane receptor molecules as a result of receptor shedding [95,96,97]. Nevertheless, to modulate HIV disease, TNF blocking agents and/or TNF inhibitor therapy could be useful, since HIV infection is characterized by immune activation and inflammation [98]. Several years ago the use of thalidomide, a TNF inhibitor, was suggested to reduce the serum TNF level and to control the viral load [99,100]. The transcription and biosynthesis of TNF is blocked by LMP-420—(2-amino-6-chloro-9-[5(dihydroxyboryl)-pentyl] purine—, a purine nucleoside analog, which inhibits the replication of HIV-1 [101]. A considerable argument in favor of the use of anti-TNF therapy in HIV-1 infected patients is the safety of its use, since no increase in the mortality rate has been observed [102]. Furthermore, reports showed that HIV-1 symptoms are improved in treated patients, even if anti-TNF therapy could favor the immunosuppressive status of the patients and thereby increase the risk of opportunistic infections [103]. Anti-TNF therapy (etanercept, infliximab, adalimumab) has been shown to be well-tolerated in HIV-infected patients, with no enhancement of the rate of opportunistic infections, unless they had uncontrolled HIV replication [104,105,106,107,108]. Anti-TNF therapy may be helpful for the treatment of autoimmune diseases, without enhancing the plasma viremia in patients whose HIV disease is under control by cART [103,109,110].

## 8. Modulation of TNF Activity to Clear HIV-1 Reservoirs

For a complete viral eradication in HIV-1 infected patients, the persistence of HIV-1 reservoirs is a major challenge [111,112,113]. Even in non-viremic patients that are under cART, the formation of an HIV-1 reservoir could result from the persistent immune activation observed especially in the presence of TNF [114]. TNF is a well-known activator of NF-κB, which is required to favor HIV-1 integration and the establishment of latency in CCL19-treated resting CD4+ T-cells [115]. Among HIV-1 reservoirs, resting or memory CD4+ T-cells are the main target [113]. Macrophages are another important HIV-1 reservoir, due to their resistance to apoptosis with sustained viral growth at low levels [116,117]. To eradicate the latent HIV-1 pool, an activation induced purging of HIV-1 in patients receiving cART has long been a proposed mechanism as a ‘shock and kill’ approach (Figure 3B) [118]. TNF has been used to reactivate HIV-1 from latently infected cells, although with limited levels of viral reactivation [119]. Although TNF consistently activates latent HIV-1 provirus in J-Lat cells, it is much less effective in primary CD4+ T-cells isolated from aviremic HIV-1 infected patients [120,121]. Moreover, toxicity associated with TNF treatment has been observed in some cases [122]. Therefore, to purge cellular reservoirs, several new drugs that target the epigenetic regulation of HIV-1 gene expression have been tested (Figure 3B). HIV-1 is synergistically reactivated from latency by a combination therapy, using both TNF and histone deacetylase inhibitors (HDACis) [123,124]. HIV-1 transcription is activated by the HDACis such as trichostatin A (TSA), trapoxin (TPX), valproic acid (VPA), and sodium butyrate (NaBut), which favor the remodeling of the single nucleosome (nuc-1) present in the HIV-1 promoter [125,126]. In view of the ‘shock and kill’ paradigm, forced HIV-1 gene expression elicited by HDACis and TNF in the presence of an effective cART regimen may lead to clearance of the pool of latently infected cells [119,125,127]. In addition to the direct effect of HDACis on HIV-1 transcription, the enhancement of TNF mediated NF-κB activation by HDACis could result in a sustained delay in IκB alpha cytoplasmic reappearance [128]. To reactivate HIV-1 from latency in resting CD4+ T-cells of HIV-infected cART-treated patients, inhibitors of histone methyltransferase (HMTIs) have also been recently shown to be efficient [129]. Synergistic activation of HIV-1 expression by HDACi and prostratin, a NF-κB inducer that stimulates TNF production, favors viral reactivation [119,130]. Disulfiram also reactivates HIV-1 from latency in parallel to increasing the production of TNF in CD4+ T-cells [131,132]. The reactivation potential of prostratin is potentiated when used in combination with P-TEFb-releasing agents such as JQ1 [133]. Combining TNF treatment with other HIV-1 reactivating drugs such as HDACis, HMTIs, and prostratin enhances HIV-1 replication and ultimately could lead to the clearance of HIV reservoirs in infected patients under cART [134].

The ‘shock and kill’ paradigm has been challenged due to significant concerns about the feasibility and possible problems of this approach [135,136]. A novel paradigm, ‘block and lock’, has emerged recently and is based on the blockade of HIV reactivation from the latency reservoir to allow the clearance of actively infected cells by the immune system [137]. The ‘block and lock’ paradigm relies on the blockade of HIV reactivation, which could be based on several approaches (Figure 3C). First, treatments that block the signaling pathways involved in HIV-1 reactivation could be useful, including, among others, inhibitors of the mammalian target of rapamycin (mTOR) complex [138] and of the AKT pathway [139,140], potentially as well as anti-TNF treatments. Anti-TNF therapy could be useful for limiting HIV-1 reactivation from latency through the inhibition of NF-κB, MAPK, and p38 pathways. Second, treatments that limit new HIV-1 integration could also, in time, diminish the size of the reservoir. In addition to inhibitors of integrase and of lens epithelium-derived growth factor (LEDGF)/p75, which limit viral integration [141,142], anti-TNF therapy could curtail the size of the HIV-1 reservoir by controlling the cellular activation. Potential reactivation of the remaining reservoir could be subsequently cleared by HIV-specific cytotoxic T-cells (CTLs) (Figure 3C). During the initial differentiation of effector cells into memory CD4+ T-cells, NF-κB activation enhances T-cell survival [143]. TNF/TNFR signaling may contribute, through its activation of NF-κB, to the establishment and maintenance of latent HIV-1 reservoirs in memory CD4+ T-cells [115]. Additionally, TNF stimulation of Nef-expressing HIV-infected T-cells inhibits CD4+ T-cell apoptosis and thereby could increase the size of the T-cell reservoir [144]. Thus, TNF-based therapy could be used to clear HIV-1 reservoirs in HIV-infected patients under cART and treated with latency reversing agents (LRA), which will reactivate a replication-competent provirus, resulting in the expression of viral proteins, such as Nef, at increased levels. In cART-treated patients, anti-TNF therapy could be an interesting approach to favor the clearance of latently infected cells by promoting their apoptosis after stimulation by LRA, in parallel to the action of HIV-specific CTLs.

## 9. Conclusions

Both immune activation and viral reservoirs are hallmarks of HIV-1 infection and involve the TNF/TNFR pathway. Thus, modulating TNF/TNFR pathway by new therapeutic approaches could limit immune activation and curtail the size of the HIV-1 reservoir in cART-treated patients with undetectable viremia, with the ultimate goal being to cure HIV-infected patients.
